# Environmental drivers of viral community composition in Antarctic soils identified by viromics

**DOI:** 10.1186/s40168-017-0301-7

**Published:** 2017-07-19

**Authors:** Evelien M. Adriaenssens, Rolf Kramer, Marc W. Van Goethem, Thulani P. Makhalanyane, Ian Hogg, Don A. Cowan

**Affiliations:** 1 Centre for Microbial Ecology and Genomics, University of Pretoria, Natural Sciences Building II, Private Bag X20, Hatfield, 0028 South Africa; 2 School of Science, University of Waikato, Hamilton, New Zealand; 3 Institute of Integrative Biology, University of Liverpool, Crown Street, Liverpool, L69 7ZB UK; 4 Polar Knowledge Canada, 170 Laurier Avenue West, Ottawa, Ontario K1P 5V5 Canada

**Keywords:** Viromics, Soil, Antarctica, Viral diversity, Viral community structure

## Abstract

**Background:**

The Antarctic continent is considered the coldest and driest place on earth with simple ecosystems, devoid of higher plants. Soils in the ice-free regions of Antarctica are known to harbor a wide range of microorganisms from primary producers to grazers, yet their ecology and particularly the role of viruses is poorly understood. In this study, we examined the virus community structures of 14 soil samples from the Mackay Glacier region.

**Methods:**

Viral communities were extracted from soil and the dsDNA was extracted, amplified using single-primer amplification, and sequenced using the Ion Torrent Proton platform. Metadata on soil physico-chemistry was collected from all sites. Both read and contig datasets were analyzed with reference-independent and reference-dependent methods to assess viral community structures and the influence of environmental parameters on their distribution.

**Results:**

We observed a high heterogeneity in virus signatures, independent of geographical proximity. Tailed bacteriophages were dominant in all samples, but the incidences of the affiliated families *Siphoviridae* and *Myoviridae* were inversely correlated, suggesting direct competition for hosts. Viruses of the families *Phycodnaviridae* and *Mimiviridae* were present at significant levels in high-diversity soil samples and were found to co-occur, implying little competition between them. Combinations of soil factors, including pH, calcium content, and site altitude, were found to be the main drivers of viral community structure.

**Conclusions:**

The pattern of viral community structure with higher levels of diversity at lower altitude and pH, and co-occurring viral families, suggests that these cold desert soil viruses interact with each other, the host, and the environment in an intricate manner, playing a potentially crucial role in maintaining host diversity and functioning of the microbial ecosystem in the extreme environments of Antarctic soil.

**Electronic supplementary material:**

The online version of this article (doi:10.1186/s40168-017-0301-7) contains supplementary material, which is available to authorized users.

## Background

The Antarctic continent is the coldest place on earth, with mean annual surface temperatures of −20 °C or less [1]. The Mackay Glacier, Ross Dependency, is a major glacial flow in Eastern Antarctica and is found to the north of the McMurdo Dry Valleys, which collectively comprise 15% of the ice-free regions on the continent. This environment is classified as a cold hyperarid desert with mineral-based permafrost soils which are largely devoid in organic matter [2, 3]. Ecosystems of cold deserts are considered simple in comparison with hot deserts due to the absence of higher plants and their large dependency on microorganisms [4]. As such, Antarctic soils are excellent model environments to investigate microbial interactions and ecosystem processes.

For bacterial communities, spatial heterogeneity in barren Antarctic soils has been found to be high, either within the same island [5], between different Dry Valleys [6], or over large latitudinal scales [7]. Apart from general soil physicochemical differences which lead to a high heterogeneity between physically separated locations [6], soil pH seems to be a main driver with certain phyla. For example, the phyla *Acidobacteria* and *Bacteroidetes* dominate extremely alkaline soils, and *Deinococcus*/*Thermus* and *Gammaproteobacteria* dominate more neutral soils, with the relative abundance of acidobacteria inversely correlated with latitude [7–9]. In soils at wetter locations or in transiently wetted soils, members of the phylum *Cyanobacteria* are more abundant [10, 11]. While cyanobacteria are considered to be the dominant primary producers in desert systems, they are only found at very low abundances in barren soils and seem to thrive more in lithic refuge niches [12, 13]. Artificial wetting and the introduction of organic matter in Dry Valley soils of different salinities have resulted in rapid bacterial community responses, in which the dominant acidobacteria and actinobacteria were replaced with proteobacteria, bacteroidetes, or firmicutes [14]. These observations suggest that extreme environment communities are vulnerable to disturbance.

The microeukaryotic diversity in Antarctic soils is made up of fungi (ascomycetes and basidiomycetes), chlorophytes, ciliates, stramenopiles, and cercozoans, as well as nematodes, tardigrades, and rotifers in wetter soils [10, 15, 16]. As with bacterial diversity, a highly variable eukaryote biodiversity has been found, related to localized environmental factors [17].

In their review on the microbiology of Antarctic Dry Valley soils, Cary and colleagues proposed a simple, two-tiered trophic model with primary producers (e.g., cyanobacteria and algae) responsible for organic matter input for heterotrophic bacteria and fungi, which are then grazed by protozoan and metazoan consumers [18]. However, this model does not take into account the potential role of viruses which have since been shown to be numerous in Antarctic soils [19, 20]. It has even been hypothesized that polar regions may be hotspots of microbial evolution due to a higher degree of viral control on these microbial communities [21].

In this study, we investigated the double-stranded DNA (dsDNA) viral communities from 14 soils sampled from ice-free areas in the Mackay Glacier region, Eastern Antarctica. Combining soil chemistry data and viral reference-dependent and reference-independent analyses, we have identified potential abiotic drivers of soil viral community diversity and analyzed patterns in biogeography.

## Methods

### Sampling

Soils from 13 distinct sites in ice-free areas of the Mackay Glacier region, north of the McMurdo Dry Valleys (DV), and one in the Taylor Valley were collected in January 2014 and 2015. For each sampling site, four aliquots of 50-g open soil (0–5 cm depth) were collected from an approximately 1 m^2^ area, stored in sterile 50-ml polypropylene Falcon tubes (Grenier, Bio-One) at below 0 °C, and transported to the laboratory before permanent storage at −80 °C.

### Soil chemistry

Soil samples were sieved on site to remove stones and analyzed for soil pH, total nitrogen, carbon, phosphorus, and major cation content (K, Na, Ca, and Mg). Element determination in soil was performed on a LECO TruSpec® Elemental Determinator by combustion analysis. X-ray fluorescence spectrometry for major cations was performed on a Philips PW1404 XRF. Soil pH was measured using a pH meter in 1:2.5 (mass to volume ratio) soil and deionized water suspensions. Physicochemical analyses were performed at CAF (Stellenbosch Central Analytical Facilities, Stellenbosch University, South Africa) using standard quality control procedures [22].

### Sample preparation to DNA sequencing

Virus-like particles (VLPs) were extracted from the 14 soil samples (in triplicate), and their DNA was isolated using an adapted protocol as described previously [23, 24]. Briefly, 15 ml of 1% potassium citrate buffer (per liter 10 g potassium citrate, 1.44 g Na_2_HPO_4_·7H_2_O, 0.24 g KH_2_PO_4_, pH 7) was added to 5 g soil, the suspensions were sonicated on ice for 3 min (30% amplitude, 1-min intervals, Qsonica sonicator), centrifuged (3000×*g*, 4 °C, 30 min) and passed through 0.45-μm cellulose acetate syringe filters (GVS) to remove remaining non-VLPs. PEG 8000 (in 1 M NaCl) was added at a final concentration of 10% (4 °C, overnight). After centrifugation and resuspension in Tris-buffer (10 mM Tris-HCl, 10 mM MgSO_4_, 150 mM NaCl, pH 7.5), remaining free DNA/RNA was digested with DNase I (Thermo Scientific, #EN0523) and RNase H (Thermo Scientific, #EN0531). Viral capsids were lysed by adding EDTA (final concentration of 20 mM), SDS (final concentration of 0.5%), and proteinase K (Thermo Scientific, #AM2546). DNA was extracted by two rounds of phenol/chloroform/isoamyl alcohol (25:24:1) and one round of chloroform/isoamyl alcohol (24:1) phase separation. The DNA was precipitated by adding 1/10 volume of sodium acetate (3 M, pH 5.0) and 2.5 volumes of ethanol (95%) and resuspended in 30 μl Milli-Q water. As a negative control, a blank sample containing 5 ml Milli-Q water instead of 5 g of soil was processed as described above.

A random priming-mediated sequence-independent single-primer amplification (RP-SISPA) approach for DNA genomes was used for amplification of the viral DNA, as previously described [25]. The following modifications to the protocol were used for amplification of SISPA primer-labeled template using the Kapa2G Robust PCR kit (Kapa Biosystems): 25 μl reactions containing 5 μl 5× reaction buffer A, 5 μl 5× reaction enhancer, 4 μl dNTPs (2.5 mM stock), 2 μl SISPA primer FR20RV, 0.5 μl MgCl_2_ (25 mM stock), 3 μl template DNA, and 0.2 μl Kapa2G Robust Taq polymerase (5 U/μl) were incubated at 95 °C for 2 min, followed by 40 cycles of 95 °C for 30 s, 58 °C for 45 s, 72 °C for 45 s, and a final extension step at 72 °C for 5 min. No reconditioning PCR was performed. For all samples (14 soil samples and one blank), libraries were constructed at the Ion Torrent Facility of the University of Pretoria (South Africa) and sequenced on three (five libraries per chip) Ion Proton Chips (PI Proton Chip, Thermo Scientific) at the platform located at Central Analytical Facilities, Stellenbosch.

### Microbial host analyses

Metagenomic sequencing was performed on the same surface soil communities as described above. Total DNA was extracted from ~2 g soil material using a 50–50–50 phenol-chloroform buffer solution described previously [26], with minor modifications [27]. Paired-end sequencing (2 × 250 bp) on an Illumina HiSeq 2500 instrument at a commercial supplier was used (Mr DNA, Shallowater, TX, USA). Raw sequences were quality filtered, trimmed, and merged using in-house scripts and PRINSEQ lite v0.20.4 [28]. Fast Length Alignment of Short reads (FLASH) was used for the alignment of forward and reverse reads [29]. All high-quality sequences were compared to the entire NCBI-nr non-redundant database using DIAMOND (BLASTX) with the sensitive option implemented at an *e* value cutoff of 1 × 10^−5^ [30]. Taxonomy was visualized in MEGAN v5.2.1 with default parameters [31]. Hits corresponding to specific taxa or functions were retained under default parameters, that is, if their bit scores were within 10% of the best bit score. All singletons were excluded from the analyses.

### In silico analyses of virome data

#### Quality control and assembly

Quality control of the reads, contamination screening, and assembly were performed with CLC Genomics Workbench 8.5.1 (CLC Bio). Initial quality control and adapter trimming of raw sequence data was based on the following parameters, quality filtering 0.05, trim first 15 bases, minimum read length 35 bases, and maximum read length 220 bases. For contamination screening, reads of the soil datasets were mapped to blank sample sequences, the human reference genome hg18 (GRCh38, UCSC Genome Browser), and an *Escherichia coli* reference genome (strain C43(DE3), GenBank accession number CP011938) to remove non-soil virus sequences. De novo assembly of each soil virome was performed with the assembly and mapping algorithm using default parameters for Ion Torrent data. Fourteen assembled viromes were uploaded to the MetaVir server (http://metavir-meb.univ-bpclermont.fr/, Project: Antarctic Soil) [32, 33].

#### Reference-independent analyses

Dinucleotide frequency analysis was performed through the MetaVir pipeline on the contig data. Cross-assembly and read-mapping was used to compare samples based on de novo cross-contig abundance profiles [34]. Briefly, post-QC reads of all 14 viromes were assembled together using CLC. The .ace file containing mapping information and individual read files were then analyzed with crAss version 2.0 [34].

#### Reference-dependent analyses

The contig datasets were analyzed through MetaVir. BLASTp (maximum *e* value of 10^−5^) was used for a comparison of the predicted genes (best BLAST hit) with the RefSeq viral protein database from NCBI (release of 2015-01-05) computing the taxonomic composition. In parallel, the post-QC read datasets were compared with the RefSeq viral protein database (downloaded from the NCBI ftp server on November 23rd 2015) using DIAMOND BLASTx (threshold of 10^−5^ for *e* value) and a maximum number of 10 hits [30]. The output was imported into MEGAN5 for taxonomic assignments with 928,976 reads assigned to known viruses in total, and then normalized to the smallest dataset (66,368 reads per sample) [31, 35]. The MEGAN5 comparison output was loaded into RStudio (Version 0.99.878, RStudio Inc., Boston, MA, USA) for statistical analyses [36, 37], which were only performed on the known fraction of reads, i.e., unassigned reads were ignored.

#### Statistics

PRIMER-E (Version 6.1.6, Plymouth Marine Laboratory, UK) was used for multivariate statistics analyses (Clarke and Warwick 2001). Hierarchical agglomerative clustering was performed for the resemblance matrices together with SIMPROF (*p* < 0.05), which tests for significant groups in an a priori unstructured data set (permutations for mean profile: 1000; simulation permutations: 999). Matrices were compared using RELATE as described previously [38]. To match biotic with environmental patterns, BEST analysis was performed (number of permutations, 99). The best subset of environmental factors was identified by calculating rho between two matrices with steadily increasing combinations of factors. All environmental data were log transformed prior to BEST.

Diversity indices were computed using the vegan (Version 2.3-3) and BiodiversityR (Version 2.6-1) packages in R [39, 40]. Correlations were calculated on relative abundance data at the family level, using Spearman’s rank correlation coefficient (rho) at *p* < 0.05 from the BiodiversityR package. Redundancy analysis (RDA) was performed in R with vegan using Hellinger transformed relative abundance data and a reduced (nine factor) soil chemistry dataset so that the number of explanatory variables was lower than the number of samples (pH, N, P, C, K, Na, Ca, Mg, altitude).

## Results

### Sampling site overview

**Fig. 1:**
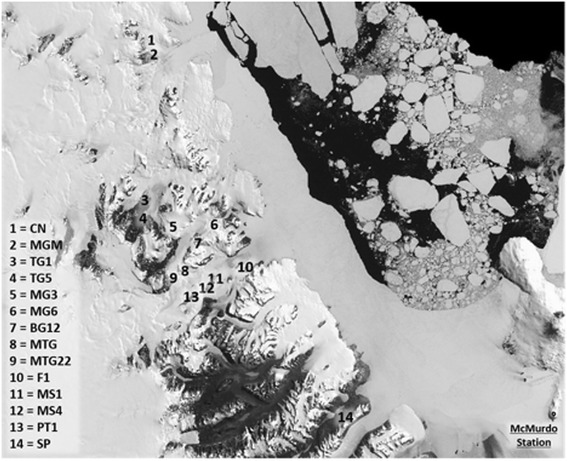
Map of the sampling region in the Dry Valley system of Antarctica (US Geological Survey, 2016, Landsat Image Mosaic Of Antarctica)

Locations of sampling sites are indicated in Fig. 1, and GPS coordinates for each site are given in Additional file 1: Table S1. Sampling sites represented different ice-free areas with different altitudes and were selected as follows: 2× Mount Seuss (MS1, MS4), 2× Benson Glacier (BG12, MG6), 2× Towle Glacier (TG1, TG5), 2× Mount Gran (MTG, MTG22), and one site each from Cliff Nunatak (CN), Flatiron (F1), Tiger Glacier (MG3), Mount Murray (MGM), Pegtop Mountain (PT1), and Spalding Pond (SP). An overview of the raw sequencing data for each site is given in Additional file 1: Table S1.

### Reference-independent analyses

**Fig. 2:**
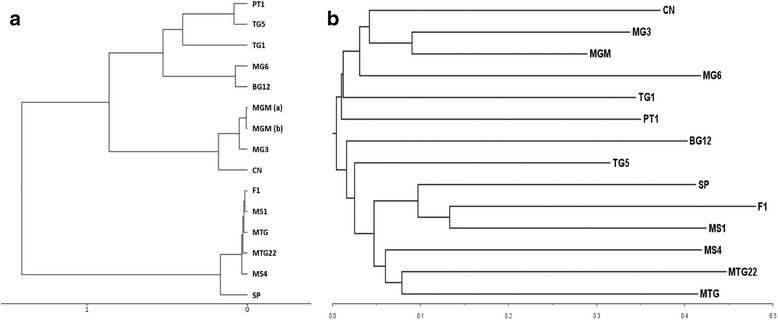
Reference-independent-based clustering of Antarctic viromes. **a** Hierarchical clustering based on dinucleotide frequencies in the contigs calculated by the MetaVir server pipeline. The *x*-axis denotes eigenvalues distances. Virome read datasets were assembled to contigs prior to analysis. Dataset MGM was split in two parts (**a, b**) due to size limitations of the pipeline. **b** crAss clustering of the 14 viromes in this study. The distances between viromes were calculated by crAss [34] using the SHOT distance formula [71], while the cladogram was created by crAss with BioNJ [72] and visualized with FigTree [73]. The branch lengths and scale axis represent the distance measures

Dinucleotide frequency and crAss analyses were used to compare the full dataset (all cleaned reads, known and unknown contigs) of the viromes of the samples. These analyses allowed for the comparison of our 14 viromes, independent of homology to reference databases such as Viral RefSeq or the non-redundant protein database of NCBI. For the dinucleotide analysis, similarity/dissimilarity of the viromes was assessed by comparing dinucleotide frequencies in all contigs. Figure 2a shows two distinct major clusters for all datasets. One cluster was composed of samples F1, MS1, MTG, MTG22, MS4, and SP. Respective dinucleotide frequency patterns exhibited high relative similarities to each other. A second cluster was composed of more distinct viromes, subdivided into two subgroups: (i) MGM (split in two subsamples), MG3 and CN and (ii) PT1, TG5, TG1, MG6, and BG12. The dinucleotide frequencies of samples MGM, MG3, and CN showed higher similarity to each other than those of the samples of subgroup PT1, TG5, TG1, MG6, and BG12. For these samples, the relative diversity in dinucleotide frequency was high in comparison to the other subgroup and cluster.

For the cross-assembly analysis, a new contig set was created by assembling all post-QC reads together. These contigs were then analyzed with the program crAss, which identified contigs present in all or a subset of the samples and produced a distance matrix which was visualized as a cladogram (Fig. 2b). The cladogram showed large branch lengths across all samples, indicating a high sequence heterogeneity in the virus communities (i.e., a low number of shared cross-assembly contigs). The viromes with the largest number of shared sequences were MGM, MG3, and CN, which clustered together. These viromes were still, however, very distinct from each other with dissimilarity values between 0.45 and 0.63 (distance matrix not shown).

### Diversity analysis

Diversity indices were calculated at the family, genus, and species level for all samples (subsampled read datasets). Accumulation plots were generated to test whether the number of viromes was sufficient to describe the full viral diversity of the Antarctic soils (Additional file 1: Figure S1). At the family level, the 14 viromes gave an adequate description of the available total viral diversity, as the curve reached an asymptote at approximately 39 families (Chao1 estimate 39.37). For the genus and species levels, the curves did not entirely reach a plateau and the total number of observed genera and species (144 and 1111, respectively) was lower than the estimated total richness (Chao1 155.61 and 1303.62, respectively). We therefore chose to make comparisons between samples based on the family-level data (Table 1).

**Table 1:** Diversity indices for Antarctic virome subsampled read datasets at the family level

Sample code	Diversity rank^a^	Diversity class	Relative abundance	Richness	Shannon-Weaver	Simpson	Fisher alpha	Beta diversity^b^
MGM	1	High	47,349	33	1.71	0.73	3.47	0.19
MG3	2	High	49,352	31	1.61	0.71	3.22	0.27
CN	3	High	48,498	29	1.57	0.70	2.99	0.36
MG6	4	Medium	52,488	21	1.04	0.45	2.07	0.87
TG1	5	Medium	55,589	27	0.85	0.35	2.72	0.46
BG12	6	Medium	55,612	18	0.91	0.38	1.73	1.19
MS4	7	Medium	61,053	19	0.55	0.26	1.82	1.07
PT1	8	Medium	60,350	20	0.35	0.13	1.93	0.97
SP	9	Medium	59,309	18	0.36	0.14	1.72	1.19
MS1	10	Medium	60,886	19	0.33	0.13	1.82	1.07
TG5	11	Medium	61,573	18	0.25	0.09	1.72	1.19
MTG	12	Low	61,433	11	0.32	0.13	1.00	2.58
MTG22	13	Low	60,981	13	0.24	0.08	1.20	2.03
F1	14	Low	60,484	14	0.20	0.07	1.30	1.81

^a^The diversity rank (1 most diverse to 14 least diverse) was calculated based on the ranking for the Shannon-Weaver, Simpson, and Fisher alpha indices

^b^Beta diversity was calculated as *β* = *S*/*α* − 1 with *α* as the alpha diversity or richness per site and *S* as the total number of families in this soil collection calculated as Chao’s estimate (*S* = 39.37)

Based on the diversity indices listed in Table 1, three classes of diversity (high, medium, and low) were defined. These were significantly different from each other, based on all indices (Kruskal-Wallis rank sum test, *p* < 0.05). In sites with high diversity (MGM, MG3, CN), more than 29 different virus families were present, representing more than 100 different genera and more than 600 species. In contrast, the low-diversity samples (MTG, MTG22, F1) had a maximum of 14 different families, containing between 23 and 58 genera and between 90 and 332 species. The beta diversity index (*β* = *S*/α − 1) was closest to zero for the high-diversity samples, as was expected, indicating that these sites were closest to approaching the full soil viral diversity at the family level.

**Fig. 3:**
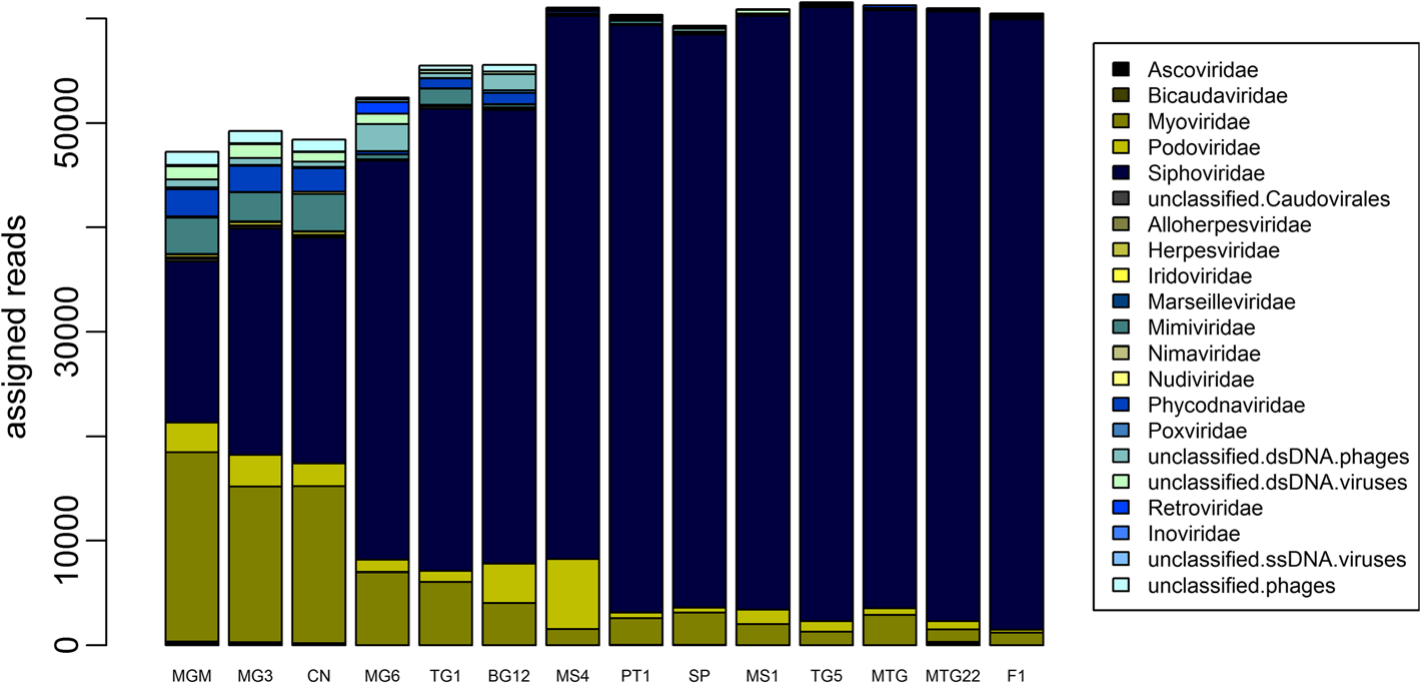
Stacked bar charted of the family-level composition of the viromes arranged according to diversity rank from most diverse to least diverse. Only families present in more than 50% of the samples are displayed

At the family level (Fig. 3), the low- and medium-diversity samples were completely dominated by *Siphoviridae* signatures, while for the high-diversity samples, this percentage was below 45%. The latter sites (MGM, MG3, and CN) contained a higher proportion of myoviruses (30–38%) and *Mimiviridae* and *Phycodnaviridae* members and other rare virus signatures mainly belonging to unclassified virus groupings. At all sites, bacteriophages belonging to the order *Caudovirales* made up more than 80% of the signatures. While, on average, the relative abundance ranking of tailed phage families was *Siphoviridae* > *Myoviridae* > *Podoviridae*, site MGM contained the family *Myoviridae* as the dominant taxon, and in site MS4, podoviruses were the second most abundant grouping behind the family *Siphoviridae*. In general, following the tailed phages, the next most common families were part of the NCLDV (nucleocytoplasmic large DNA virus) grouping, *Mimiviridae*, members of which infect amoeba, and *Phycodnaviridae*, with members infecting different types of unicellular algae. With a total of 38 observed families in the entire dataset, the majority of these fall into the rare fraction, being present at low abundances and in only a subset of the sampling sites. Grouped by host, these rare families infect bacteria (*Tectiviridae*, *Microviridae*, *Inoviridae*), archaea (*Bicaudaviridae*, *Lipothrixviridae*, *Rudiviridae*), unicellular eukaryotes (*Marseilleviridae*), insects (*Ascoviridae*, *Asfarviridae*, *Baculoviridae*, *Malacoherpesviridae*, *Iridoviridae*, *Nimaviridae*, *Nudiviridae*, *Poxviridae*, *Hytrosaviridae*), vertebrates (*Alloherpesviridae*, *Herpesviridae*, *Circoviridae*, *Picornaviridae*, *Polyomaviridae*, *Poxviridae*, *Retroviridae*), and plants (*Caulimoviridae*, *Potyviridae*).

Making genus-level comparisons of viral communities is more difficult than at the family level, because the taxonomy of virus genera is not yet comprehensive, i.e., many species are only classified to the family level. Therefore, the most abundant genus-level classifications for these viromes were “unclassified *Siphoviridae*” (80% of total dataset), “unclassified *Myoviridae*” (5%), and “unclassified *Podoviridae*” (2%). Notwithstanding the “unclassified” and “unassigned” groupings, the three most abundant genera of siphoviruses were *Lambdavirus*, *Barnyardvirus*, and *Spbetavirus*. For the *Myoviridae*, the broad host-range genera *T4virus*, *P2virus*, and *Bxz1virus* were most commonly identified, while the most abundant genera of podoviruses included *N4virus*, *Phikmvvirus*, and *P22virus*. The majority of these genera have members which infect common soil bacteria such as *Mycobacterium smegmatis*, *Bacillus* spp., and *Pseudomonas* spp. or are genera which contain a high number of published genome sequences. Other genera present at more than 0.2% in the total dataset were the phycodnavirus genera *Chlorovirus*, *Prymnesiovirus*, and *Prasinovirus* and the mimivirus genus *Cafeteriavirus*.

At the species level, 23% of total read assignments (summed over all datasets) were attributed to two *Rhodococcus* phages, ReqiPepy6 and ReqiPoco6, which are unclassified *Siphoviridae* showing 75% identity at the DNA level [41]. The third most commonly identified virus species was *Bacillus* phage G, a giant bacteriophage with a genome of 497.51 kb, currently an unassigned singleton species within the *Myoviridae* family (RefSeq accession number NC_023719).

For assembled contigs, identification of the taxonomic composition at the family was achieved by analyzing contig datasets with MetaVir. For all samples, significant reference database hits varied between 4.7 and 18.7% of the predicted genes (Median = 11.7%), of which 96–98% were identified as dsDNA viruses. The order *Caudovirales* was dominant in all viromes with a median of 82.5% of the significant hits (range = 62–92%). Samples MGM (62%), CN (62%), and MG3 (64%) exhibited the lowest values, correlating with the read composition. In MTG22, MS1, MTG, F1, SP, MS4, and TG5, the proportion of predicted genes affiliated with the order *Caudovirales* was found to be more than 82.5%.

### Co-occurrence networks

**Fig. 4:**
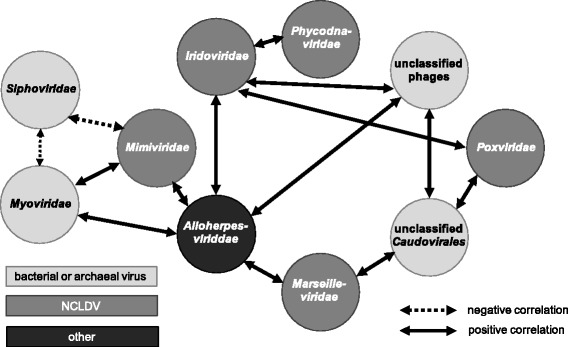
Co-occurrence diagram of the viral families showing significant correlations across the 14 viromes (Spearman rank order, *p* < 0.05)

Diversity analysis demonstrated an inverse correlation between the incidence of siphoviruses and other families (Fig. 3). Although the presence of members of the *Siphoviridae* family was not directly correlated with any of the diversity indices presented in Table 1 (Spearman rho, *p* > 0.05), we did find significant correlations between relative abundances of different families. These are visualized as a co-occurrence network (Fig. 4). The incidence of members of the family *Siphoviridae* was negatively correlated with those of the families *Myoviridae* and *Mimiviridae*. The occurrence of these families was in turn positively correlated with each other and a network of positively interacting families (only significant correlations shown). This network grouped viruses which either belonged to the grouping NCLDV or prokaryotic viruses. The family *Alloherpesviridae* groups viruses of fish and amphibians and was only found in very low abundance, and its presence in the network was probably an artifact of this.

### Abiotic drivers of Antarctic soil viral community composition

**Fig. 5:**
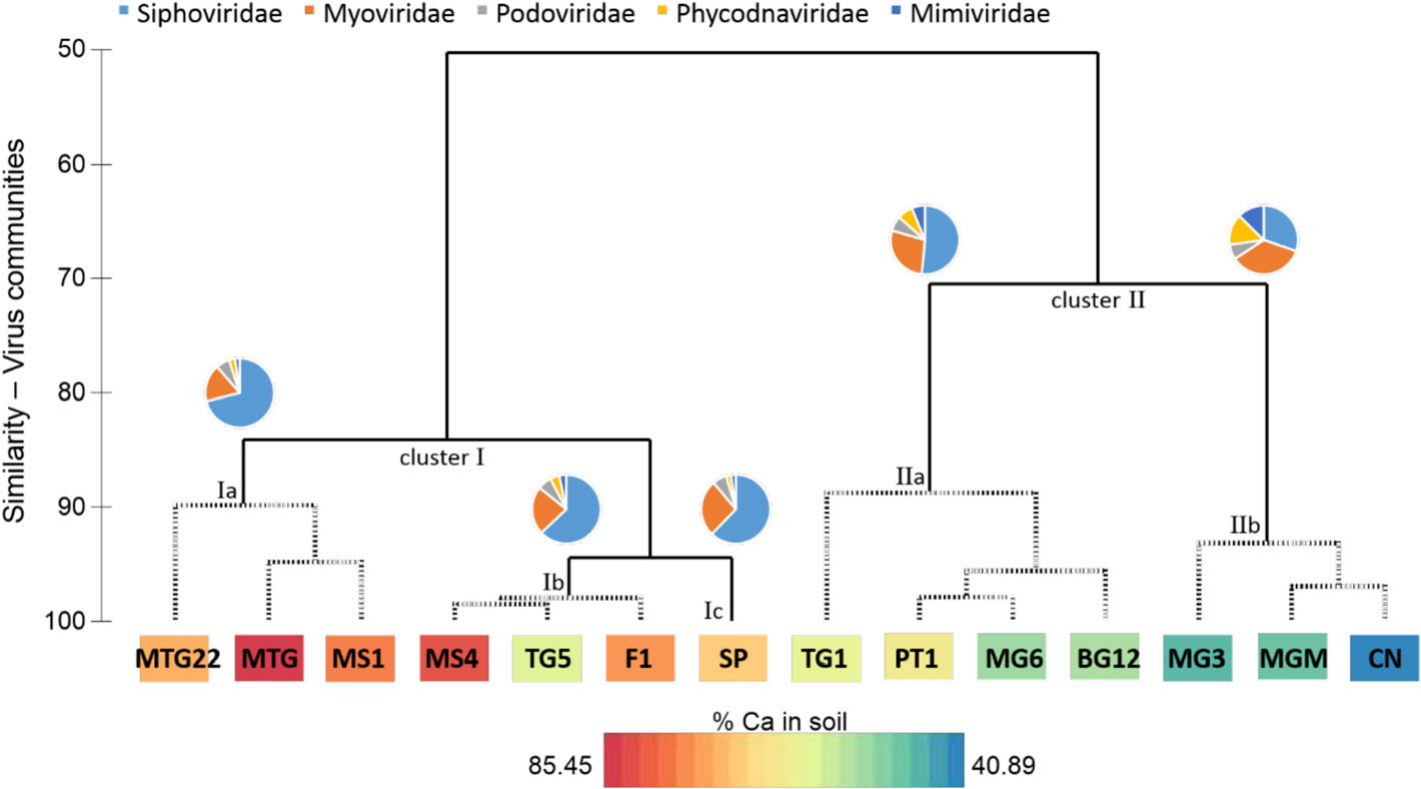
Hierarchical agglomerative clustering based on the Bray-Curtis similarity matrix of soil virus communities. The complete linkage mode was used, and simprof was performed to identify two clusters and five distinct subgroups which are indicated with *dashed lines*. For each subgroup, a *pie chart* shows the relative abundance of the five virus families. The individual color for each family is shown on top. A color gradient indicates the relative proportion of calcium (% Ca) for each soil samples. Minimum and maximum values (in %) are shown with the gradient at the bottom

To investigate the effect of environmental factors on the dominant virus genotypes, samples were clustered according to the individual community structure of their viromes (Fig. 5) before correlation analyses were applied. Contig datasets were analyzed with abundant virus families being defined as those having more than 1% of significant hits. In total, six different virus families were considered: *Siphoviridae*, *Myoviridae*, *Podoviridae* (all belonging to the order *Caudovirales*), *Phycodnaviridae*, *Mimiviridae*, and *Poxviridae*. Poxviruses were only abundant in samples MGM, CN, and MG3 (range = 1–1.5%), but the remaining families were abundant in all samples (Additional file 1: Table S2). By calculating a resemblance matrix using the Bray-Curtis similarity coefficient (scale 0 to 100 from least to most similar), the community composition of all viromes was compared and clustered (Fig. 5). Two major clusters emerged from this analysis. Cluster I, composed of MTG22, MTG, MS1, MS4, TG5, F1, and SP, was subdivided into subgroups Ia, Ib, and Ic at a high similarity value of 85, while subgroups of cluster II were more dissimilar to each other and subdivided at a similarity value of 70. In total, five distinct subgroups were confirmed by SIMPROF. For each distinct subgroup, a pie chart (see Fig. 5) indicated the average relative abundance of all five consistently abundant virus families. For the subgroups from left to right, relative abundance of *Siphoviridae* members decreased whereas those of *Myoviridae* increased (corresponding with the results of the co-occurrence analysis in Fig. 4). Similarly, the relative abundances of *Phycodnaviridae* members increased from 2 to 4% relative abundance in MTG22, MTG, and MS1 (subcluster Ia) to 12–13% in MGM, CN, and MG3 (subcluster IIb). On the contrary, the relative abundance of podoviruses was comparatively constant across all samples (5–8%).

**Fig. 6:**
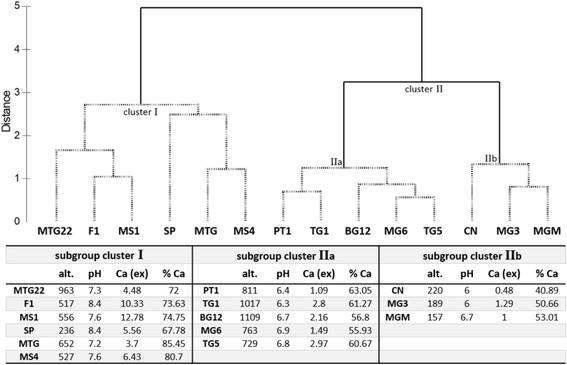
Environmental drivers found by multivariate correlation analysis. Identified drivers were altitude (alt.), pH, calcium (ex; exchangeable cations), and % Ca. *Top*: hierarchical agglomerative clustering based on the Euclidean distance matrix using identified environmental drivers only. Untransformed values of the factors were normalized prior to analysis. The group average mode was used, and simprof was performed to identify two distinct subgroups indicated with *dashed lines*. *Bottom*: table with individual values of environmental drivers for each subgroup and sample

To analyze and compare the viral community composition with respect to environmental factors, 15 environmental factors (14 soil chemistry properties and the sampling site altitude) were determined (Additional file 1: Table S3). A two-level approach was used for the analyses: linear correlation analysis with single environmental factors (superimposed in Fig. 5) and multivariate correlation analysis with multiple environmental factors (Fig. 6). Using linear correlations, the soil pH and calcium content (% Ca) were identified as significant abiotic drivers of the most abundant viral genotypes (Fig. 5). All samples in cluster II had a soil pH of between 6 and 7, while all samples of cluster I had a pH above 7 (except for sample TG5). As illustrated by the color gradient in Fig. 5, the percentage of calcium in the soil (in relation to all other major cations) was positively correlated with the presence of siphoviruses (Pearson *R* = 0.850) and negatively correlated with phycodnavirus signatures (Pearson *R* = −0.767). Using a multivariate statistical approach to find a combination of multiple environmental factors best explaining the observed viral taxonomic composition, a combination of four environmental factors was identified: sampling site altitude, soil pH, soil calcium content as exchangeable cations, and the percentage of soil calcium compared with the other major cations (rho = 0.649, *p* = 0.01). All samples were clustered according to these four environmental factors (Fig. 6), and this clustering was compared with the taxonomy-based clustering (Fig. 5) to test if the four-factor model adequately explained the differences in viral composition between samples. In Figs. 5 and 6, cluster I and II as well as subgroups IIa and IIb comprised the same samples (with the exception of sample TG5). In addition, the validity of this model was confirmed by performing the RELATE procedure of PRIMER-E (rho = 0.537, *p* = 0.01). In conclusion, for the most abundant families, the drivers of viral community composition were pH, calcium content (% Ca as well as exchangeable cations), and sampling site altitude.

**Fig. 7:**
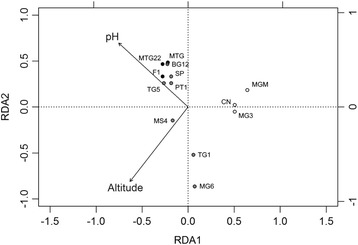
Redundancy analysis of the family-level viral community compositions of the 14 soil read datasets. *Dots* for the high-diversity samples are filled in *white*, for medium diversity in *gray*, and low diversity in black. The family relative abundance data was Hellinger transformed and the soil parameters were reduced to independent nine factors. RDA1 explained 65.2% of the variation while RDA2 explained 0.7% of the variation. Only the parameters with a significant impact, pH, and altitude, are shown (RDA permutation test, pH *p* = 0.01, altitude *p* = 0.035)

In an alternative approach to investigating environmental drivers of soil virus communities, the complete family-level composition of the read datasets (abundant and rare fraction) was used in a redundancy analysis (RDA). For this analysis, the soil chemistry dataset was reduced to nine variables, excluding interdependent parameters (Additional file 1: Table S3). Only two variables (soil pH and site altitude) showed a significant impact on the complete taxonomic composition (Fig. 7). A distinct grouping, comprising the low-altitude, high-diversity sites MGM, MG3, and CN was observed in the RDA plot, corresponding with subclusters in Figs. 5 and 6. Samples from these three sites had pH values between 6.0 and 6.7 and were located at low elevations (156–220 m above sea level). In this constrained analysis, RDA1 explained 65.2% of the difference between samples and RDA2 only 0.7%, clustering samples MG6 and TG1 separately, and the other medium- and low-diversity samples in a third cluster. Sites MG6 and TG1 (medium diversity) were located at higher altitudes (763 and 1017 m, respectively) and had a soil pH of below 7. RDA of the contig dataset yielded the same significant variables (soil pH and site altitude) and a similar pattern of clustering, clearly separating the high-diversity samples from the others (data not shown).

Analyses of both contig and read datasets clearly identified soil pH and sampling site altitude as the dominant abiotic drivers of viral community composition, while the soil calcium content contributed to the distribution of the most abundant genotypes, particularly for the families *Siphoviridae* and *Phycodnaviridae*.

### Host microbiome diversity

The microbial diversity of the soil samples was assessed by metagenomic analysis of total microbial DNA extracted from the same sites (Van Goethem et al., unpublished results). These soils were dominated by bacteria, making up ~79% of the sequences. Eukaryotes and Archaea were considerably less common with ~21 and 0.05%, respectively, of taxon-assigned sequences belonging to these groups. The high-diversity sites, MGM, MG3, and CN, showed a considerably higher abundance of archaeal and eukaryotic signatures than the lower diversity sites (Additional file 1: Table S4). Consistent with the viral results presented here, microbial community structure was significantly driven by site altitude. Overall, the soil microbial communities were dominated by four bacterial phyla, *Acidobacteria*, *Bacteroidetes*, *Proteobacteria*, and *Chloroflexi*, which accounted for more than half of the total microbial sequences.

## Discussion

An immediate observation from analysis of Antarctic soil virus communities is that the samples showed high sequence heterogeneity, independent of geographical locations (illustrated by the branch lengths in Fig. 2b). Samples taken from the Mount Seuss sites (MS1 and MS4), physically separated from each other by less than 2 km and 30 m altitude, showed high sequence dissimilarity. None of the dissimilarity values in the crAss analysis exceeded 0.87, implying that a core virus component was shared between all samples, albeit a minor fraction compared with the sample-specific virus community. This confirms the results of a fingerprinting analysis on similar Antarctic soils, where virus communities were found to be significantly different over distances as short as 20 m [42]. It is argued that the discrete nature of soil particles and micro-aggregates present in undisturbed soils can lead to resource specialization of microbial communities and can thus lead to greater community diversification at micro-spatial scales [43, 44]. The impact of localized environmental factors is, therefore, expected to be greater than general geographical effects, as previously observed in temperate soil bacterial communities [45].

A comparison was made of the most abundant taxa in these soil viromes with other polar viromes (Antarctic and Arctic regions, Table 2). The imported soil viromes were the most similar to the soil viromes in this study. The family distribution of *Siphoviridae > Myoviridae > Podoviridae > Phycodnaviridae > Mimiviridae* was largely conserved, except for the virome investigated by GeoChip microarray analysis [46]. In the latter study, a significant number of tectivirus signatures were found in some of the soils, at levels comparable with tailed phages. This can most likely be explained by the difference in sampling strategy and phage lifestyles. The known soil tectiviruses are all temperate phages which would have been identified in a marker-based analysis of total nucleic acid, but would not be recovered in our study, where DNA was extracted of extracellular viral particles. At the genus level, there were more differences, but the dominant genera were conserved over the different studies. The viral communities in the previously described water-based polar viromes are much less similar to the 14 soil viromes of this study. Both in Antarctic lakes and Arctic glaciers, single-stranded DNA (ssDNA) viruses are the dominant group of the viral communities. Within this group, environmental sequences classified in the family *Circoviridae* are the most abundant. Two studies showed a seasonal spike in phycodnavirus presence, most likely due to the increased abundance of their algal hosts in summer [47, 48].

**Table 2:** Comparison of the most abundant taxa identified in selected polar viromes

Virome	Sample type	Most abundant families	Most abundant genera^a^	Reference
Antarctic soil comparison	Soil	*Siphoviridae*, *Myoviridae*, *Podoviridae*, *Phycodnaviridae*, *Mimiviridae*	*Lambdavirus*, *T4virus*, *N4virus*, *Chlorovirus*, *Cafeteriavirus*	This study
Antarctic soil and hypolith	Soil	*Siphoviridae*, *Myoviridae*, *Podoviridae*, *Phycodnaviridae*, *Mimiviridae*	*Lambdavirus*, *T4virus*, *Epsilon15virus*, *Chlorovirus*, *Cafeteriavirus*	[19]
Antarctic soil and hypolith	Hypolith	*Siphoviridae*, *Myoviridae*, *Podoviridae*, *Phycodnaviridae*, *Mimiviridae*	*P70virus*, *T4virus*, *Sp6virus, Chlorovirus*, *Moumouvirus*	[19]
Antarctic lithic niche GeoChip	Soil	*Siphoviridae*, *Tectiviridae*, *Myoviridae*, *Podoviridae*, *Leviviridae*, *Microviridae*	–	[46]
Lake Limnopolar (Antarctica) spring	Water	*Circoviridae*, *Microviridae*, *Nanoviridae*, *Siphoviridae*	Unclassified environmental ssDNA viruses, unclassified lambda-like viruses	[47]
Lake Limnopolar (Antarctica) summer	Water	*Phycodnaviridae*, *Circoviridae*, *Mimiviridae*, *Myoviridae*, *Siphoviridae*	*Prasinovirus*, *Moumouvirus*, unclassified ssDNA viruses, *T4virus*, *E125virus*	[47]
Antarctic meromictic lake	Water	*Phycodnaviridae*, *Siphoviridae*, *Myoviridae*, *Podoviridae*	*-*	[48]
Arctic cryoconite	Water	*Siphoviridae*, *Myoviridae*, *Podoviridae*, *Phycodnaviridae*, *Mimiviridae*	*P70virus*, *T4virus*, *Bcep22virus*, *Chlorovirus*, *Moumouvirus*	[74]
Arctic freshwater	Water	*Circoviridae*, *Microviridae, Geminiviridae*, *Siphoviridae*	-	[75]

^a^The first most abundant genus in the families in the previous column. Data from previous publications were extracted from MetaVir, where possible

The discrepancy in the presence and abundance of ssDNA viruses between the different studies is, in our view, due to two reasons. The first one is technical and attributed to the phi29-based multiple displacement amplification (MDA) of viral DNA used in the other studies, versus the use of RP-SISPA in our study. MDA preferentially amplifies circular ssDNA viruses leading to an overrepresentation of this group in the final datasets [49–51]. In contrast, RP-SISPA is biased towards the most abundant viruses, as well as those with the largest genomes, and will therefore underrepresent small or less abundant ssDNA viruses [25, 52]. Other technical factors in the extraction and analysis protocols will have introduced additional biases. For example, the use of filters at different pore sizes, PEG precipitation or ultracentrifugation, differing library preparation methods and sequencing platforms, may complicate comparisons between studies. Bioinformatic analyses can also contribute to underestimation of ssDNA viruses, which have genomes orders of magnitude smaller than those of the most abundant tailed phages found in this study (~5 kb versus 50–200 kb complete genome length, respectively). Therefore, a similar number of ssDNA virus and tailed phage genomes will contribute different numbers of sequencing reads to the dataset which can bias read-based analyses. Our additional analysis of assembled reads into contigs, which confirmed our findings, was performed to limit some of the bioinformatics-based bias. The second reason is likely due to the differences in sampling type, soil versus water, and most significantly differences in host populations between edaphic and aquatic habitats. It is possible that ssDNA viruses are not a significant component of the soil microbiome, whereas they represent major biotic components of aquatic environments. With our processing method, we cannot, however, confirm this statement and additional studies need to be performed specifically investigating the presence (relative abundance) or absence of ssDNA viruses in soils.

The dominant abiotic drivers of viral taxonomic composition were identified as pH, calcium content, and sampling site altitude. A combination of different factors, rather than linear correlations with individual factors, was found to best explain the community composition, which probably reflects the heterogeneity of Antarctic soils [3]. Physical and chemical properties of DV soils are found to vary in close geographic location [53, 54], possibly explaining the patchiness of soil biota [55]. Soil pH has been previously found to be a good predictor of microbial community structure over large spatial scales [45, 56]. In DV soils, calcium content has also been reported to significantly impact bacterial population structures [57]. The effect these soil parameters have on viral communities is, therefore, probably best explained by the influence they have on host community composition.

The effect of sampling site altitude is most probably linked to temperature and water availability and has been previously observed for nematodes at low-altitude DV soils [55]. The mean summer temperatures of surface soils at low-altitude Dry Valleys (elevations below 400 m: the coastal thaw zone) are typically around 2 °C, whereas higher altitude valleys have mean temperatures below 0 °C during the same period (−5 °C for the stable upland zone) [58]. In permafrost soils of the high-altitude University Valley (1700 m above sea level), the mean summer temperature of surface soils was −23 °C and no active microbial metabolism could be observed [59]. However, these soils still contained a highly diverse bacterial community comprised mainly of the phylum *Proteobacteria* and differing levels of *Firmicutes*, *Actinobacteria* and *Bacteroidetes* [59]. The altitude-dependent temperature regime of surface soils directly influences microbial metabolism and is expected to substantially impact viral communities, as these require metabolically active hosts for their replication. We therefore hypothesize an underlying basis for altitude as driver of viral taxonomic composition via the effect of temperature on microbial (host and virus) metabolic rates.

Several significant trends were observed in viral community composition patterns (beta diversity). Our analyses showed that increasing diversity and higher family richness negatively correlated with the incidence of members of the family *Siphoviridae* (Figs. 3 and 4). Bacteriophages of the order *Caudovirales* were dominant in all samples, but the presence of members of the families of *Myoviridae* and *Siphoviridae* showed an inverse correlation. We hypothesize that siphoviruses and myoviruses are direct competitors for hosts in the same niche. Our results indicate that siphoviruses are present at higher relative abundances in high-calcium-content soils with neutral to alkaline pH and in the low host-metabolism environment present at higher altitudes (Figs. 5 and 6). This could be explained by the finding that siphoviruses are the dominant type of prophages in bacterial genomes [60], and lysogeny employed by temperate phages can act as refuge strategy under environmental conditions which are unfavorable for the host [61]. This is supported, albeit indirectly, by the fact that the high-diversity viromes have a lower incidence of predicted integrase genes (data not shown). The bacteriophages of the *Podoviridae* family, in contrast, were present at similar levels in all samples and showed no correlations with other families or soil parameters. Consequently, we suggest that members of this family are not in direct competition with other phages for hosts. Whether this is due to differences in host specificity or the use of different infection strategies remains unclear. Given the presence of these viruses at similar levels throughout the sample set, one can infer that podoviruses may infect members of the phyla which were present at constant levels in all samples, such as *Bacteroidetes*, *Proteobacteria*, or *Acidobacteria*. In general, the host microbial data showed differences in the presence of bacterial phyla in the soil samples. However, linking these differences specific bacterial taxa to infecting viruses is complex and error-prone with bioinformatics methods less than 40% accurate [62].

Sampling sites with the highest virus diversity showed a relatively high abundance of members of the families *Phycodnaviridae* and *Mimiviridae* (both members of the NCLDV grouping), which infect unicellular eukaryotes. The same distribution was found for the host microbial communities, with high-diversity samples harboring a higher abundance of eukaryotes and a lower abundance of bacterial hosts. The phycodnaviruses, mimiviruses, and other NCLDV families are shown as an interconnected network of co-occurring viruses (Fig. 4), suggesting viruses belonging to these families may not compete for hosts.

The two-tiered trophic model [18] can now be expanded with a third tier, comprised of viruses involved in a “bottom-up” trophic regulation of the soil microbial communities [46]. This model has been widely accepted for marine microbial communities where lysis through the “viral shunt” is responsible for a significant proportion of elemental cycling with an increase in organic nutrients [63–65]. A recent marine model even predicts that steady-state coexistence of bacterial and eukaryotic autotrophs is impossible without viral lysis [64]. If this model is applied to Antarctic soils, it provides a partial explanation for the presence of a positively correlated network of bacteriophages (myoviruses, unclassified tailed phages) and eukaryotic viruses (NCLDVs, mainly *Mimiviridae* and *Phycodnaviridae*), implying a combination of these viruses is necessary to maintain the presence of the hosts. With respect to the highly variable taxonomic composition of viruses in Antarctic soils, we hypothesize that the impact of soil viruses on their hosts is more variable than in the marine environment. For example, the widespread incidence of lysogeny in soils [66, 67] can limit the contribution of soil viruses to organic nutrient release via host lysis but may have a beneficial effect on hosts through lysogenic conversion [68].

## Conclusions

Many studies have laid the groundwork for understanding Antarctic biodiversity (reviewed previously [69]), but the role of viruses in soil ecosystems remains very poorly understood. This is the first in-depth metagenome sequencing-based study of open Antarctic soils focusing on virus diversity and the influence of environmental factors. We have identified soil pH and sampling site altitude as the two main drivers for viral community composition in our datasets. Additionally, in combination with calcium content of the soil, these factors best explain the most abundant viral family distribution. We have also speculated on the correlations between different viral families in this type of model environment, finding a potentially intricate web of inter-family interactions impacting each other and the host environment.

Future challenges include linking soil viruses to their cognate hosts [62, 70], exploring functional contributions of viruses to ecosystem functioning, and incorporating their interactions and ecological functions into soil ecosystem models.

## Additional file

Additional file 1: Table S1. Sample overview. Coordinates of sampling sites and sequencing metadata of viromes. Cleaned reads = reads after removal of contaminants; reads for assembly = reads considered by the assembly and mapping algorithm of CLC Genomics Workbench at the default parameters. **Table S2.** Taxonomic diversity of the assembled viromes. Diversity was assessed by MetaVir and abundant virus families defined as representing more than 1% of significant hits. All values are given as relative abundances in percent. Values for sample MGM, which needed to be split in two parts for upload to MetaVir, represent both parts (a and b; individual values for the separate parts are shown at the bottom). Sign. hits = significant reference database hits of the predicted genes at a maximum *e* value of 10^−5^. Families belonging to the order *Caudovirales* are marked with an asterisk. **Table S3.** Environmental parameters measured for all soil samples. ex cat = exchangeable cations, variables designated with an asterisk were used in the RDA. **Table S4.** Microbial diversity in samples as determined by metagenomic sequencing, as percentage of the total reads assigned. **Figure S1.** Accumulation plots of the different taxa present in the 14 Antarctic virome read datasets. A) family level, B) genus level, C) species level.

## Abbreviations

(RP-)SISPA: (Random priming-mediated) sequence-independent single-primer amplification
BG12/MG6: Benson glacier
BLAST: Basic Local Alignment Search Tool
CN: Cliff Nunatak
DV: McMurdo Dry Valleys
F1: Flatiron
MDA: Multiple displacement amplification
MG3: Tiger glacier
MGM: Mount Murray
MS1/MS4: Mount Seuss
MTG/MTG22: Mount Gran
NCLDV: Nucleo-cytoplasmic large DNA virus
PT1: Pegtop Mountain
QC: Quality control
RDA: Redundancy analysis
SP: Spalding pond
TG1/TG5: Towle glacier
VLP: Virus-like particle
