# Supplementary material for: Environmental drivers of viral community composition in Antarctic soils identified by viromics
Source: Microbiome. 2017 Jul 19;5:83. doi: 10.1186/s40168-017-0301-7 (PMC5518109; doi:10.1186/s40168-017-0301-7)
Supplement: Additional file 1: Table S1. — Sample overview. Coordinates of sampling sites and sequencing metadata of viromes. Cleaned reads = reads after removal of contaminants; reads for assembly = reads considered by the assembly and mapping algorithm of CLC Genomics Workbench at the default parameters. Table S2. Taxonomic diversity of the assembled viromes. Diversity was assessed by MetaVir and abundant virus families defined as representing more than 1% of significant hits. All values are given as relative abundances in percent. Values for sample MGM, which needed to be split in two parts for upload to MetaVir, represent both parts (a and b; individual values for the separate parts are shown at the bottom). Sign. hits = significant reference database hits of the predicted genes at a maximum e value of 10−5. Families belonging to the order Caudovirales are marked with an asterisk. Table S3. Environmental parameters measured for all soil samples. ex cat = exchangeable cations, variables designated with an asterisk were used in the RDA. Table S4. Microbial diversity in samples as determined by metagenomic sequencing, as percentage of the total reads assigned. Figure S1. Accumulation plots of the different taxa present in the 14 Antarctic virome read datasets. A) family level, B) genus level, C) species level. [file 40168_2017_301_MOESM1_ESM.docx]

# Supplementary information

Table S1: Sample overview. Coordinates of sampling sites and sequencing metadata of viromes. Cleaned reads = reads after removal of contaminants; Reads for assembly = Reads considered by the assembly and mapping algorithm of CLC Genomics Workbench at the default parameters

| Sample | GPS coordinates | Raw sequence reads | Cleaned reads | Average length of reads in bases | Reads for assembly | N50 |
| --- | --- | --- | --- | --- | --- | --- |
|  |  | **Reads after initial quality check** | **Reads left (of raw reads)** |  | **Final contigs** | **Average length** |
| BG12 | 76° 52.144' S | 21 186 415 | 2 131 862 | 176.16 | 2 011 228 | 433 |
|  | 161° 45.148' E | 19 314 468 | 10% |  | 8 015 | 406bp |
| MG6 | 76° 49.358' S | 20 123 508 | 8 361 261 | 160.16 | 7 789 892 | 471 |
|  | 162° 06.422' E | 19 654 614 | 42% |  | 24 841 | 435bp |
| MS1 | 77° 01.149' S | 16 944 481 | 9 328 473 | 174.11 | 8 755 127 | 543 |
|  | 161° 46.931' E | 14 938 075 | 55% |  | 24 061 | 473bp |
| F1 | 77° 00.377' S | 20 912 606 | 14 975 200 | 168.89 | 13 817 390 | 532 |
|  | 162° 23.841' E | 20 316 625 | 72% |  | 22 097 | 490bp |
| MG3 | 76° 47.005' S | 19 301 644 | 15 628 026 | 173.1 | 11 903 828 | 314 |
|  | 161° 27.014' E | 17 413 725 | 81% |  | 337 278 | 312bp |
| CN | 76° 06.627' S | 20 610 904 | 14 353 168 | 160.15 | 11 972 177 | 485 |
|  | 162° 00.839' E | 19 768 349 | 70% |  | 217 972 | 462bp |
| TG5 | 76° 43.731' S | 21 623 308 | 15 944 417 | 165.27 | 13 940 456 | 349 |
|  | 161° 00.699' E | 21 002 972 | 74% |  | 84 180 | 355bp |
| MS4 | 77° 01.384' S | 24 273 428 | 12 418 070 | 167.48 | 11 433 750 | 430 |
|  | 161° 42.794' E | 23 761 023 | 51% |  | 23 870 | 404bp |
| SP | 77° 39.003' S | 19 270 177 | 13 429 500 | 165.51 | 11 708 257 | 348 |
|  | 163° 07.001' E | 18 727 685 | 70% |  | 47 939 | 351bp |
| TG1 | 76° 39.277' S | 23 267 783 | 16 415 556 | 166.39 | 14 861 145 | 467 |
|  | 161° 05.577' E | 22 665 576 | 71% |  | 67 554 | 427bp |
| MTG | 76° 57.133' S | 17 917 775 | 3 120 837 | 151.33 | 2 869 110 | 505 |
|  | 161° 24.263' E | 17 386 811 | 17% |  | 2 879 | 450bp |
| MGM | 76° 09.646' S | 16 556 234 | 15 392 604 | 162.94 | 8 610 550 | 392 |
|  | 162° 00.950' E | 15 979 557 | 93% |  | 624 603 | 383bp |
| PT1 | 77° 02.762' S | 22 599 479 | 12 919 279 | 165.93 | 11 635 296 | 471 |
|  | 161° 21.559' E | 21 920 115 | 57% |  | 56 018 | 442bp |
| MTG22 | 76° 58.027' S | 20 876 313 | 16 969 909 | 162.65 | 15 589 391 | 532 |
|  | 161° 09.730' E | 20 365 928 | 81% |  | 9 322 | 481bp |

Table S2: Taxonomic diversity of the assembled viromes. Diversity was assessed by MetaVir and abundant virus families defined as representing more than 1% of significant hits. All values are given as relative abundances in percent. Values for sample MGM, which needed to be split in two parts for upload to MetaVir, represent both parts (a & b; individual values for the separate parts are shown at the bottom). Sign. hits = significant reference database hits of the predicted genes at a maximum e value of 10^-5^. Families belonging to the order *Caudovirales* are marked with an asterisk.

| Sample | Sign. hits | dsDNA viruses | *Caudo virales* (*) | * *Sipho viridae* | * *Myo viridae* | * *Podo viridae* | *Phycodna viridae* | *Mimi viridae* | *Pox viridae* |
| --- | --- | --- | --- | --- | --- | --- | --- | --- | --- |
| BG12 | 10.98 | 96 | 78 | 49 | 22 | 6 | 5 | 5 | 0 |
| MG6 | 11.53 | 97 | 80 | 47 | 25 | 7 | 6 | 4 | 0 |
| MS1 | 20.94 | 98 | 90 | 62 | 18 | 8 | 2 | 2 | 0 |
| F1 | 14.44 | 97 | 85 | 58 | 20 | 5 | 4 | 3 | 0 |
| MG3 | 4.73 | 97 | 64 | 28 | 28 | 7 | 12 | 10 | 1 |
| CN | 7.9 | 97 | 62 | 24 | 32 | 5 | 12 | 11 | 2 |
| TG5 | 12 | 98 | 85 | 57 | 20 | 7 | 4 | 3 | 0 |
| MS4 | 13.63 | 98 | 85 | 57 | 22 | 6 | 4 | 3 | 0 |
| SP | 12.73 | 98 | 88 | 56 | 24 | 6 | 2 | 2 | 0 |
| TG1 | 8.76 | 97 | 72 | 40 | 26 | 6 | 8 | 8 | 1 |
| MTG | 17.4 | 98 | 89 | 66 | 18 | 5 | 4 | 3 | 0 |
| MGM | 4.79 | 97 | 62 | 25 | 30 | 6 | 13 | 11 | 1.5 |
| PT1 | 10.96 | 98 | 80 | 47 | 26 | 6 | 7 | 5 | 0 |
| MTG22 | 18.7 | 98 | 92 | 72 | 13 | 5 | 2 | 1 | 0 |
| - | - | - | - | - | - | - | - | - | - |
| MGM (a) | 5.41 | 97 | 62 | 25 | 30 | 6 | 13 | 11 | 1 |
| MGM (b) | 4.16 | 97 | 61 | 25 | 30 | 6 | 13 | 11 | 2 |

Table S3: Environmental parameters measured for all soil samples. ex cat = exchangeable cations, variables designated with an asterisk were used in the RDA.

| Sample | Altitude (m)* | pH* | P (mg/kg)* | K (mg/kg)* | C %* | N %* | Na (ex cat)* | K (ex cat)* | Ca (ex cat)* | Mg (ex cat)* | Tvalue (cmol/kg) | Na % | K % | Ca % | Mg % |
| --- | --- | --- | --- | --- | --- | --- | --- | --- | --- | --- | --- | --- | --- | --- | --- |
| BG12 | 1109 | 6.7 | 11 | 61 | 0.1 | 0.02 | 0.6 | 0.15 | 2.16 | 0.89 | 3.8 | 15.67 | 4.08 | 56.8 | 23.45 |
| MG6 | 763 | 6.9 | 18 | 83 | 0.12 | 0.016 | 0.33 | 0.21 | 1.49 | 0.63 | 2.67 | 2.52 | 7.92 | 55.93 | 23.64 |
| MS1 | 556 | 7.6 | 22 | 116 | 0.15 | 0.032 | 1.92 | 0.3 | 12.78 | 2.1 | 17.1 | 11.23 | 1.73 | 74.75 | 12.29 |
| F1 | 517 | 8.4 | 20 | 299 | 0.12 | 0.042 | 1.95 | 0.77 | 10.33 | 0.99 | 14.03 | 13.87 | 5.46 | 73.63 | 7.04 |
| MG3 | 189 | 6 | 9 | 35 | 0.1 | 0.02 | 0.13 | 0.09 | 1.29 | 0.78 | 2.54 | 5.24 | 3.53 | 50.66 | 30.75 |
| CN | 220 | 6 | 12 | 26 | 0.14 | 0.016 | 0.07 | 0.07 | 0.48 | 0.32 | 1.18 | 5.67 | 5.57 | 40.89 | 26.7 |
| TG5 | 729 | 6.8 | 11 | 27 | 0.12 | 0.019 | 0.28 | 0.07 | 2.97 | 1.57 | 4.89 | 5.75 | 1.43 | 60.67 | 32.16 |
| MS4 | 527 | 7.6 | 17 | 56 | 0.12 | 0.021 | 0.37 | 0.14 | 6.43 | 1.02 | 7.97 | 4.65 | 1.8 | 80.7 | 12.85 |
| SP | 236 | 8.4 | 33 | 220 | 0.12 | 0.022 | 1.21 | 0.56 | 5.56 | 0.88 | 8.2 | 17.7 | 6.85 | 67.78 | 10.67 |
| TG1 | 1017 | 6.3 | 12 | 29 | 0.12 | 0.028 | 0.07 | 0.07 | 2.8 | 1.62 | 4.56 | 1.64 | 1.61 | 61.27 | 35.48 |
| MTG | 652 | 7.2 | 15 | 22 | 0.1 | 0.024 | 0.13 | 0.06 | 3.7 | 0.44 | 4.33 | 3.01 | 1.29 | 85.45 | 10.25 |
| MGM | 157 | 6.7 | 25 | 32 | 0.12 | 0.032 | 0.34 | 0.08 | 1 | 0.47 | 1.88 | 17.79 | 4.28 | 53.01 | 24.92 |
| PT1 | 811 | 6.4 | 4 | 15 | 0.12 | 0.008 | 0.07 | 0.04 | 1.09 | 0.53 | 1.72 | 3.83 | 2.2 | 63.05 | 30.91 |
| MTG22 | 963 | 7.3 | 19 | 75 | 0.1 | 0.028 | 0.32 | 0.19 | 4.48 | 1.37 | 6.72 | 4.77 | 2.87 | 72 | 20.36 |

Table S4: Microbial diversity in samples as determined by metagenomic sequencing, as percentage of the total reads assigned.

| Sample | Bacteria | Eukaryotes | Archaea |
| --- | --- | --- | --- |
| MGM | 80.4 | 19.6 | 0.00 |
| MG3 | 69.0 | 31.0 | 0.00 |
| CN | 71.6 | 28.2 | 0.16 |
| MG6 | 72.3 | 27.7 | 0.06 |
| TG1 | 82.9 | 17.1 | 0.00 |
| BG12 | 85.9 | 13.9 | 0.15 |
| MS4 | 80.0 | 19.9 | 0.03 |
| PT1 | 87.4 | 12.6 | 0.02 |
| SP | 72.0 | 28.0 | 0.00 |
| MS1 | 84.8 | 15.1 | 0.15 |
| TG5 | 85.3 | 14.6 | 0.06 |
| MTG | 83.5 | 16.5 | 0.00 |
| MTG22 | 76.2 | 23.7 | 0.12 |
| F1 | 71.9 | 28.1 | 0.00 |


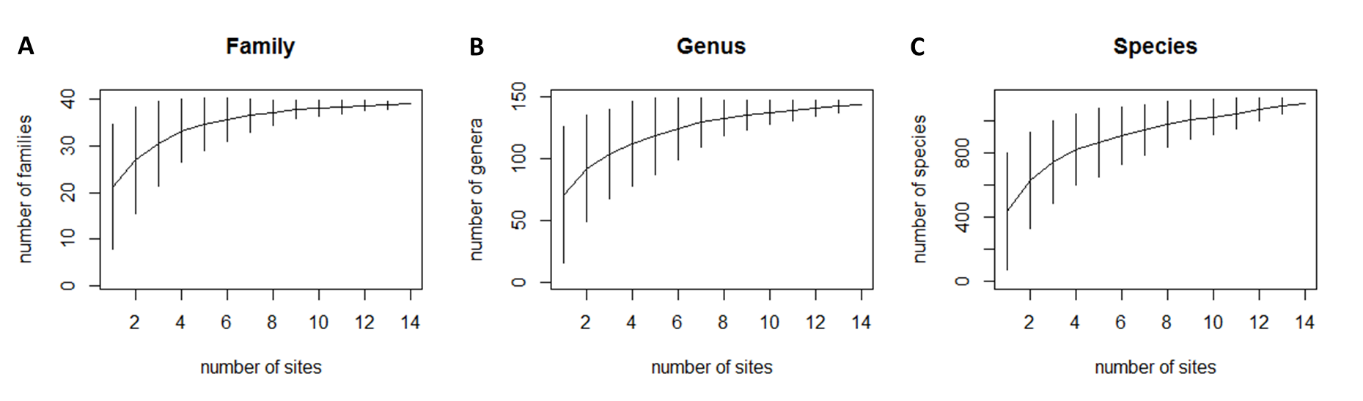


Figure S1: Accumulation plots of the different taxa present in the 14 Antarctic virome read datasets. A) family level, B) genus level, C) species level.
